# Beyond lifespan and healthspan: a biological framework for experienced longevity

**DOI:** 10.3389/fnagi.2026.1815030

**Published:** 2026-04-07

**Authors:** Tomas Liubertas

**Affiliations:** Wellness Institute, Vilnius, Lithuania

**Keywords:** aging, arterial stiffness, episodic memory, event segmentation, mitochondrial function, neurovascular coupling, nitric oxide, retrospective time perception

## Abstract

Aging research has traditionally focused on lifespan and healthspan as primary outcome domains, implicitly treating chronological time as a uniform container of value. The memory-structured reconstruction of extended chronological intervals has not been systematically examined as an aging-related variable. I introduce *experienced longevity*, defined as the amount of lived time subjectively contained within a fixed chronological interval. Drawing on established distinctions between retrospective and prospective duration judgments, I argue that long-interval temporal compression reflects memory-structured reconstruction rather than altered internal timekeeping. Building on event segmentation theory, I propose that experiential density - the number and distinctiveness of retrievable experience units per unit time - determines whether extended intervals are remembered as compressed or expanded. I present the Neuroenergetic Constraint Model, which posits that aging-related reductions in mitochondrial efficiency, increased vascular stiffness, and diminished nitric oxide–mediated neurovascular coupling constrain neuroenergetic flexibility. Reduced energetic reserve may limit high-fidelity updating during ongoing experience, weaken event segmentation, decrease experiential density, and increase the probability of retrospective temporal compression. The model generates falsifiable predictions linking biological markers of neuroenergetic reserve (e.g., metabolic efficiency, arterial stiffness, endothelial function) to segmentation performance and retrospective duration judgments, particularly under high-demand conditions. If supported, this framework expands aging science beyond survival and function to include the biological structuring of lived time.

## 1 Introduction

Aging research has traditionally focused on two primary outcome domains: lifespan, the length of survival, and healthspan, the duration of life lived in good functional health. These metrics have driven major advances in biogerontology and translational aging science (Rattan, 2018; Max Planck Institute for Biology of Ageing, 2026). However, both treat chronological time as a fixed unit of value. A year of chronological time is typically assumed to represent comparable experiential encoding across individuals and age groups. This assumption is rarely examined.

Yet subjective reports across adulthood consistently suggest that extended periods - months and years - are often remembered as having “passed quickly,” particularly in later life. Importantly, this phenomenon does not imply a change in objective time but reflects differences in how time is encoded and reconstructed (Martinelli and Droit-Volet, 2022; Droit-Volet et al., 2018). Time-perception research distinguishes moment-to-moment passage-of-time judgments from retrospective duration judgments, and evidence indicates that long-interval judgments rely heavily on memory structure rather than internal clock mechanisms (Martinelli and Droit-Volet, 2022; Droit-Volet et al., 2018; Block and Zakay, 1997). *This raises a testable possibility: aging may influence not only survival and functional capacity, but the density of retrievable episodic content within a fixed chronological interval.*

I introduce the concept of experienced longevity, defined as the amount of lived time subjectively contained within a fixed chronological interval. Within the present framework, this construct is operationalized through experiential density, defined as the number and distinctiveness of event units segmented, encoded, and later retrievable per unit of chronological time. I propose that age-related biological changes - particularly declines in mitochondrial efficiency (Gainutdinov et al., 2023; Steffan et al., 2025; Somasundaram et al., 2024; Lesnefsky and Hoppel, 2006), increased vascular stiffness (Mitchell, 2021; El Assar De La Fuente et al., 2012; Vatner et al., 2021), and reduced nitric oxide- mediated neurovascular coupling (Hoiland et al., 2020; Lourenço and Laranjinha, 2021; Ledo et al., 2021; Lourenço et al., 2017) may constrain the brain’s capacity for high-fidelity updating during ongoing experience. By limiting event segmentation and episodic distinctiveness, these neuroenergetic constraints may increase the probability of retrospective temporal compression (Martinelli and Droit-Volet, 2022; Droit-Volet et al., 2018; Block and Zakay, 1997; Hu et al., 2025).

I term this framework the Neuroenergetic Constraint Model of experienced longevity. In this framework, experienced longevity is the broader aging-related construct, experiential density is the proximate memory-level property through which it is expressed, and retrospective temporal compression is the downstream subjective outcome expected when that density is reduced.

## 2 Cognitive foundations of retrospective temporal compression

### 2.1 Retrospective duration judgments are memory-structured

Research on subjective time consistently distinguishes passage-of-time judgments (“How fast does time feel right now?”) from duration judgments (“How long was that interval?”) (Martinelli and Droit-Volet, 2022; Droit-Volet et al., 2018; Block and Zakay, 1997). Empirical work shows that these measures can dissociate and that age differences depend strongly on which construct is assessed (Martinelli and Droit-Volet, 2022; Mioni et al., 2021; El Haj et al., 2013). Critically, retrospective duration judgments - particularly for extended real-world intervals such as weeks or months - depend more heavily on memory-related processes than on prospective attention-to-time mechanisms (Martinelli and Droit-Volet, 2022; Block and Zakay, 1997; Zakay and Block, 2004).

Meta-analytic evidence supports a long-standing distinction: prospective timing relies primarily on attentional allocation, whereas retrospective timing relies more strongly on the structure and availability of encoded memory traces (Block and Zakay, 1997; Zakay and Block, 2004). For long intervals, individuals reconstruct duration based on what is retrievable rather than on continuous internal timekeeping (Block and Zakay, 1997; Zakay and Block, 2004; Droit-Volet and Wearden, 2016; Droit-Volet et al., 2017).

This memory dependence provides a concrete mechanism for long-interval temporal compression. When fewer distinct episodic elements are encoded and later accessible, extended periods are more likely to be reconstructed as shorter.

Importantly, alterations in retrospective duration judgments are not limited to normative aging. Neurodegenerative conditions, including Alzheimer’s disease and related disorders, have also been associated with disturbances in temporal awareness and duration estimation, suggesting that disruptions in neural systems supporting memory and contextual processing may alter subjective time perception (Mioni et al., 2021; El Haj et al., 2013).

Conversely, subjective time compression is not exclusive to older adults. Younger individuals may also experience similar distortions under conditions of elevated stress or high cognitive load, which can reduce contextual encoding and thereby influence retrospective duration judgments (Block et al., 2010).

### 2.2 Experiential density and event segmentation

Continuous experience is not encoded as an undifferentiated stream. Event segmentation research demonstrates that individuals naturally parse ongoing activity into discrete events and subevents, often hierarchically. Event boundaries are associated with updating processes and play a critical role in organizing memory representations (Swallow et al., 2009; Shim et al., 2024).

Within this framework, I define experiential density as the number and distinctiveness of event units that are segmented and encoded during ongoing experience and remain available for later retrieval per unit of chronological time. This construct is not metaphoric; it is grounded in the memory dependence of retrospective duration judgments and in established findings on event segmentation and memory updating (Martinelli and Droit-Volet, 2022; Block and Zakay, 1997; Mioni et al., 2021; El Haj et al., 2013; Zakay and Block, 2004; Shim et al., 2024).

Higher experiential density - characterized by frequent, well-defined event boundaries and distinctive episodic encoding - should increase the amount of retrievable content associated with a given interval. Lower experiential density - characterized by fewer or weaker boundaries and reduced distinctiveness - should increase the probability of retrospective temporal compression (Shim et al., 2024; Flores et al., 2017; Kurby and Zacks, 2008).

Importantly, experiential density is not determined solely by novelty or lifestyle factors. It depends on the brain’s capacity to dynamically update representations during ongoing experience. This observation opens a mechanistic question: what biological constraints might limit the fidelity of event segmentation and episodic encoding across aging (Lourenço et al., 2017; Hu et al., 2025; Mioni et al., 2021; El Haj et al., 2013)?

It is important to distinguish experiential density from distortions that arise during later memory reconstruction. In the present framework, experiential density refers primarily to the density of event units that were originally segmented and encoded during ongoing experience, not to details that may be added retrospectively through schema-based inference or false recollection. Accordingly, memory distortions may inflate the *apparent* density of experience when this construct is estimated solely from delayed recall, but they do not imply that more event boundaries were originally encoded. If anything, such distortions may blur the remembered distinctiveness of adjacent events and thereby complicate retrospective estimates of experiential structure (Swallow et al., 2009; Flores et al., 2017; Jeunehomme and D’Argembeau, 2020; Brewer and Treyens, 1981; Roediger and McDermott, 1995).

## 3 Neuroenergetic constraints in aging

### 3.1 Neural computation requires dynamic energetic support

Neural processing is metabolically demanding. The maintenance of ion gradients, synaptic transmission, and network-level integration require continuous availability of adenosine triphosphate (ATP) together with tightly regulated substrate delivery (Attwell and Laughlin, 2001; Harris et al., 2012). The brain therefore relies on coordinated neuroenergetic and neurovascular mechanisms to match moment-to-moment neural activity with oxygen and glucose supply.

Neurovascular coupling (NVC) refers to the process by which local neural activation induces localized increases in cerebral blood flow to meet metabolic demand. Nitric oxide (NO) has been repeatedly identified as a key mediator in this coupling mechanism (Lourenço et al., 2017; Iadecola, 2017), linking glutamatergic signaling to vascular responses and thereby regulating activity-dependent perfusion. Effective cognitive processing therefore depends not only on neural architecture but also on the integrity of metabolic production and activity-to-flow matching.

Within this framework, adaptive updating during ongoing experience - including the formation of event boundaries and episodic encoding - can be understood as operations that require dynamic energetic support (Swallow et al., 2009).

High-fidelity updating under changing environmental demands is not computationally free; it depends on sufficient neuroenergetic flexibility. Synaptic transmission and synaptic plasticity are themselves energetically demanding. Experimental work shows that synaptic vesicle cycling imposes substantial ATP requirements, particularly during endocytosis (Pathak et al., 2015), and that metabolic support is required for long-term memory formation for hippocampal long-term potentiation (Suzuki et al., 2011). Because hippocampal synaptic plasticity is essential for memory acquisition (Tsien et al., 1996), reduced flexibility of energy supply may be expected to impair encoding-related operations that depend on contextual updating, associative binding, and the maintenance of distinct event representations.

### 3.2 Aging and reduced neuroenergetic flexibility

Aging is associated with multiple biological changes that plausibly constrain this flexibility.

First, mitochondrial function declines across adulthood, with evidence for impairments in oxidative phosphorylation efficiency, altered dynamics, and increased oxidative stress. Because neurons are highly energy-dependent, reductions in mitochondrial efficiency may limit cellular energetic reserve, particularly under conditions of increased demand (Gainutdinov et al., 2023; Steffan et al., 2025; Somasundaram et al., 2024; Lesnefsky and Hoppel, 2006; Chistiakov et al., 2014; Kowald, 2001; Crescenzo et al., 2015).

Second, vascular stiffening increases with age, reflecting structural remodeling of elastic arteries and altered hemodynamics. Reduced vascular elasticity may impair the adaptability of cerebral perfusion, particularly during rapid shifts in neural activity (Mitchell, 2021; El Assar De La Fuente et al., 2012; Vatner et al., 2021; Hu et al., 2025; Castelli et al., 2023; Herzog et al., 2025).

Third, NO bioavailability declines with age and is implicated in endothelial dysfunction and vascular aging. Given the central role of NO in NVC, reductions in NO signaling may weaken the precision of activity-to-flow matching (Mazuryk et al., 2024; Pourbagher-Shahri et al., 2021; Adhikari et al., 2025; Herrera et al., 2010; Seals et al., 2011).

**FIGURE 1:**
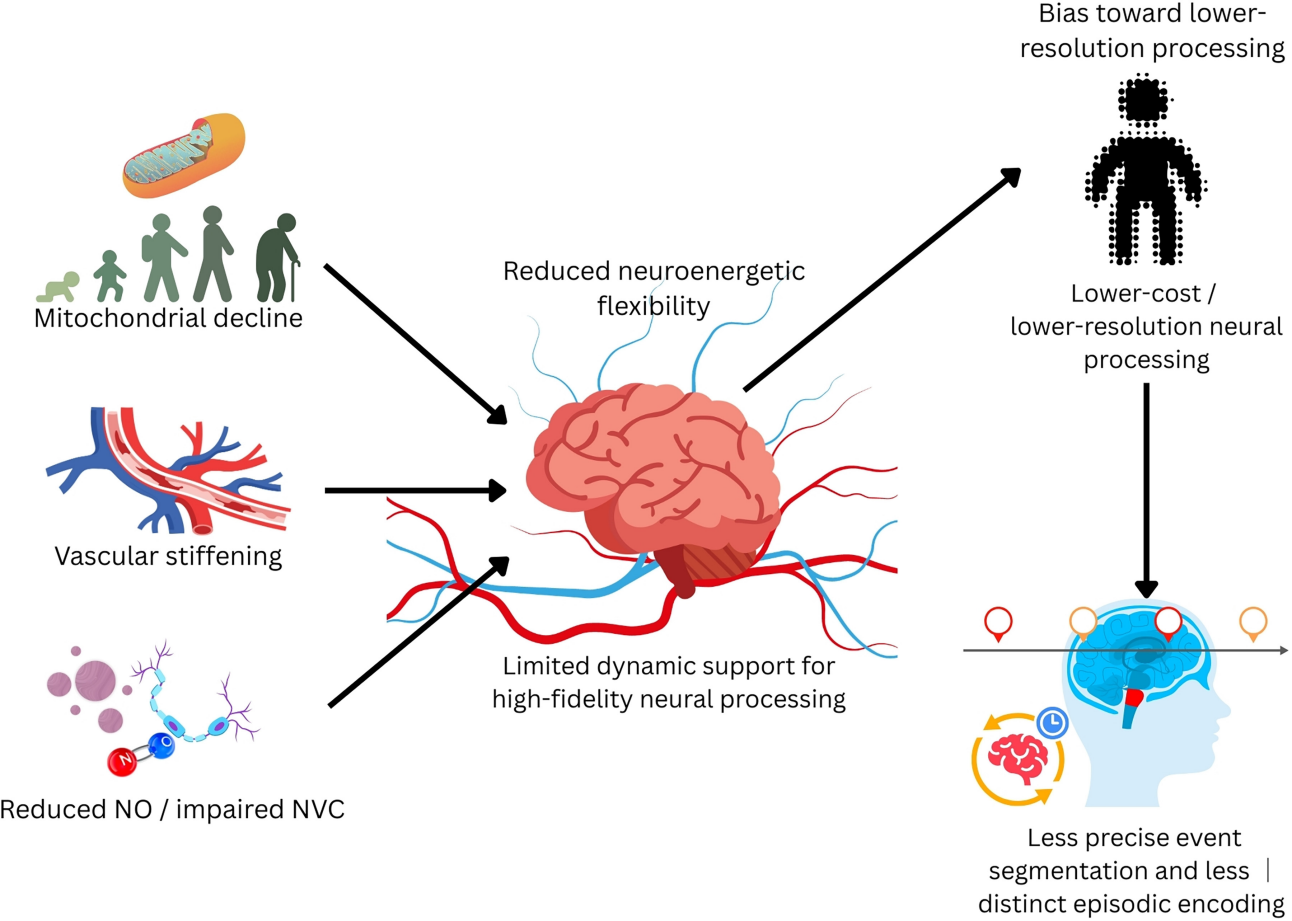
Converging age-related biological constraints on neuroenergetic flexibility. Neural updating depends on dynamic energetic support and precise activity-to-flow matching. With aging, mitochondrial decline, vascular stiffening, and reduced nitric oxide (NO)-dependent neurovascular coupling converge to reduce neuroenergetic flexibility, i.e., the capacity to sustain high-fidelity neural processing under fluctuating demand. This constraint may bias neural systems toward lower-resolution, lower-cost processing and thereby weaken event segmentation and episodic distinctiveness.

These converging changes summarized in Figure 1 suggest a broader principle: aging may reduce neuroenergetic flexibility - the capacity to dynamically sustain high-resolution neural processing under fluctuating demand. Importantly, these alterations are not restricted to normative aging. Related mitochondrial dysfunction (Somasundaram et al., 2024) and neurovascular dysregulation (Ledo et al., 2021), including impaired NO-dependent coupling (Zhu et al., 2022), have also been implicated in pathological brain aging, particularly neurodegenerative and vascular cognitive disorders (He and Sun, 2025), suggesting that the proposed neuroenergetic constraints may extend beyond normative aging.

### 3.3 From energetic constraint to experiential structure

The present framework does not propose that aging directly “accelerates time.” Rather, it suggests that reduced neuroenergetic flexibility may bias neural processing toward lower-cost operating modes. High-resolution neural processing characterized by continuous contextual updating and fine-grained event differentiation requires substantial metabolic energy (Attwell and Laughlin, 2001). When metabolic resources are constrained, cortical systems may preserve overall responsiveness while sacrificing coding precision, resulting in broader tuning and reduced fine-grained discrimination (Padamsey et al., 2022). In dynamic environments, sensory systems also adapt their input-output relations to changing stimulus statistics in ways that help optimize information transmission (Brenner et al., 2000).

If high-fidelity updating becomes more energetically constrained, the formation of distinct event boundaries may weaken, and episodic representations may become less differentiated (Swallow et al., 2009; Shim et al., 2024; Kurby and Zacks, 2008). Over extended intervals, such reductions in segmentation fidelity and episodic distinctiveness would decrease experiential density. Under memory-based models of retrospective timing, this pattern increases the probability of temporal compression (Block and Zakay, 1997; Mioni et al., 2021; El Haj et al., 2013; Zakay and Block, 2004).

This argument establishes the biological component of the Neuroenergetic Constraint Model: aging-related changes in mitochondrial function, vascular elasticity, and NO-mediated coupling may probabilistically constrain neural processes involved in event segmentation and episodic encoding.

This perspective is also consistent with broader theories of neural energy allocation. For example, the Allostatic Triage Model (Kelley et al., 2025) suggests that under conditions of constrained energetic resources, biological systems may prioritize immediate survival-relevant processes over energetically costly functions that support longer-term optimization. Although that framework was developed primarily in the context of stress-related psychopathology, it offers a useful conceptual parallel for the present model: reduced neuroenergetic flexibility may likewise bias cognitive processing toward lower-cost modes, potentially at the expense of fine-grained episodic updating.

## 4 The neuroenergetic constraint model of experienced longevity

The preceding sections establish two premises relevant to the present framework. First - retrospective duration judgments over extended intervals depend primarily on memory structure and event segmentation rather than on continuous internal timekeeping (Martinelli and Droit-Volet, 2022; Block and Zakay, 1997; Mioni et al., 2021; El Haj et al., 2013; Zakay and Block, 2004; Swallow et al., 2009; Shim et al., 2024; Flores et al., 2017). Second - aging is associated with reductions in neuroenergetic flexibility, including declines in mitochondrial efficiency, reduced vascular adaptability, and impaired NO-mediated neurovascular coupling.

### 4.1 Sequential mechanisms of the neuroenergetic constraint model

**FIGURE 2:**
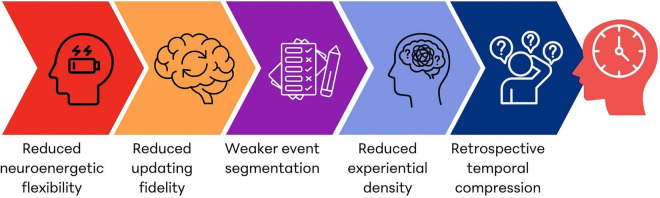
Neuroenergetic constraint model of experienced longevity. The proposed model suggests that age-related reductions in neuroenergetic flexibility may constrain the dynamic support required for high-fidelity neural updating. Reduced updating fidelity may weaken event segmentation processes and lead to lower experiential density, defined as the number of distinct episodic representations formed during a given period. When retrospective duration judgments rely on episodic memory density, reduced experiential density may increase the probability of retrospective temporal compression, contributing to the subjective acceleration of time with age.

The Neuroenergetic Constraint Model links these premises through a sequential chain of probabilistic dependencies summarized in Figure 2. The following steps are presented as a conceptual progression rather than a deterministic sequence, and their expression may vary depending on regional energetic conditions, task demands, and compensatory mechanisms.

#### 4.1.1 Step 1: reduced neuroenergetic flexibility

Aging-related declines in mitochondrial efficiency, vascular adaptability, and NO-mediated neurovascular coupling reduce the capacity to dynamically sustain high-fidelity neural activity under fluctuating demand (Gainutdinov et al., 2023; Mitchell, 2021; El Assar De La Fuente et al., 2012; Vatner et al., 2021; Hoiland et al., 2020; Lourenço et al., 2017; Stern et al., 2023). This does not imply overall cognitive impairment; rather, it suggests reduced neuroenergetic reserve - that is, reduced capacity to sustain high-fidelity neural processing under fluctuating demand - and diminished adaptive responsiveness (Harris et al., 2012).

#### 4.1.2 Step 2: reduced updating fidelity during ongoing experience

When neuroenergetic reserve is constrained, the precision with which ongoing perceptual and contextual changes are registered and integrated into evolving event models may decline, especially in complex, rapidly changing, or multitask environments. This effect should be understood probabilistically rather than deterministically: some functions may remain relatively preserved, but updating may become slower, noisier, or more selective when local demand outstrips flexible supply (Harris et al., 2012; Zhu et al., 2022).

#### 4.1.3 Step 3: weakened event segmentation and episodic distinctiveness

Reduced updating fidelity is expected to make event boundaries less precise and episodic traces less differentiated, rather than abolishing segmentation altogether. In operational terms, event segmentation can be quantified by the number, timing, and intersubject agreement of perceived boundaries during continuous activity, for example in movie-based segmentation tasks. Because boundary processing supports later memory organization, less precise updating should reduce the structural differentiation of encoded experience (Shim et al., 2024; Flores et al., 2017; Kurby and Zacks, 2008).

#### 4.1.4 Step 4: reduced experiential density

As segmentation becomes coarser and episodic traces less differentiated, the number and distinctiveness of retrievable experience units per unit of elapsed time should decline. In operational terms, experiential density may be quantified as the density of recalled experience units, or recalled moments of experience, per unit of actual event duration. Existing work on temporal compression in episodic memory suggests that this density closely tracks the grain size of event segmentation (Flores et al., 2017; Jeunehomme and D’Argembeau, 2020; Jeunehomme et al., 2022).

#### 4.1.5 Step 5: increased probability of retrospective temporal compression

Under memory-based models of retrospective duration judgments, lower experiential density increases the likelihood that extended intervals will be reconstructed as shorter (Block and Zakay, 1997; Mioni et al., 2021). The subjective impression that months or years “passed quickly” thus reflects a downstream consequence of constrained experiential structuring rather than altered objective time.

The constructs proposed in this framework can be operationalized using established paradigms from the event perception and autobiographical memory literature. Event segmentation is typically quantified using boundary detection tasks in which participants indicate perceived transitions during continuous activity, allowing measurement of boundary frequency, timing, and inter-observer agreement (Kurby and Zacks, 2008; Wahlheim and Zacks, 2025). Experiential density may be estimated retrospectively through structured recall protocols in which narratives are parsed into discrete “experience units,” with density indexed relative to the actual duration of the encoded episode (Jeunehomme and D’Argembeau, 2020).

### 4.2 Boundary conditions

The model predicts that the effect of neuroenergetic constraint on retrospective compression will be strongest under conditions in which environmental complexity and updating demands are high, contextual change is subtle or weakly marked, and neuroenergetic reserve is relatively low. For example, high updating demands may occur in situations such as hosting a large family gathering, where multiple conversations, logistical demands, and unexpected changes must be coordinated simultaneously. Conversely, contextual change may be weakly marked during extended periods spent in environments such as hospitals or care facilities, where daily routines are repetitive and externally structured. Similarly, the influence of neuroenergetic constraint may be amplified when neuroenergetic reserve is reduced, as may occur in late-life individuals with lower cardiovascular fitness or reduced metabolic flexibility who are engaged in cognitively demanding activities.

Conversely, strong segmentation cues, high emotional salience, or highly structured goal-directed engagement may partially compensate for energetic constraints by externally scaffolding boundary formation.

In this framework, neuroenergetic reserve refers to the capacity to sustain cognitive and neural processing under increased energetic demand and may be indirectly indexed by measures such as mitochondrial energetic capacity, cardiorespiratory fitness (e.g., VO_2_max), muscle strength, or metabolic flexibility (Atamna et al., 2018; Stern et al., 2023; Mau et al., 2023).

The framework would be seriously challenged if indirect markers of neuroenergetic reserve, such as mitochondrial energetic capacity, cardiorespiratory fitness (VO2max), muscle strength, and metabolic flexibility, predict retrospective compression but show no association with event segmentation or episodic distinctiveness; if observed compression effects are fully explained by affective variables, such as depression or anxiety, without contribution from markers of neuroenergetic reserve; or if no interaction emerges between neuroenergetic reserve and environmental demand.

Such outcomes would suggest that psychological or affective mechanisms are sufficient to account for retrospective temporal compression, thereby challenging the central premise that neuroenergetic constraint plays a meaningful role in shaping the subjective structure of long-term lived time.

## 5 Discussion

The Neuroenergetic Constraint Model is a theoretical integration and does not provide direct causal evidence. Each link in the proposed chain - from neuroenergetic reserve to updating fidelity, from event segmentation to experiential density, and from experiential density to retrospective compression - requires empirical validation.

First, subjective time is multidetermined, reflecting the combined influence of affective state, attentional engagement, motivational context, and neuroenergetic factors. Although the present model focuses on memory-structured retrospective compression, biological associations must be tested while controlling for affective and contextual variables.

Second, retrospective duration judgments reflect reconstructive processes. Compression effects may arise from retrieval-stage biases rather than encoding-stage segmentation differences. Experimental designs that independently measure segmentation during experience and memory structure at retrieval will be required to isolate mechanism.

Third, the biological markers proposed (e.g., arterial stiffness, endothelial function, metabolic proxies) are indirect indices of neuroenergetic flexibility. Associations could reflect broader health status rather than specific neurovascular coupling constraints. Multimodal designs combining vascular, metabolic, and neurocognitive measures will be necessary to increase specificity.

Finally, event segmentation is treated as a primary bridge mechanism, but it may not exhaustively account for experiential density. Emotional salience, narrative coherence, and goal hierarchies may independently influence the remembered structure of time.

These limitations do not invalidate the model but define the empirical work required to test it rigorously.

## 6 Conclusion

Aging may alter not only how long life continues and how well biological systems function, but also how densely life is structured in memory. Framed this way, experienced longevity is not a metaphorical extension of aging science, but a testable proposal about how biological constraint may shape the remembered architecture of lived time.

The present paper proposes experienced longevity as a complementary construct for aging science, defined as the density of retrievable experience units within a fixed chronological interval. It further advances the Neuroenergetic Constraint Model, which suggests that aging-related reductions in mitochondrial efficiency, vascular adaptability, and nitric oxide-mediated neurovascular coupling may reduce neuroenergetic flexibility, weaken event segmentation and episodic distinctiveness, and thereby increase the likelihood of retrospective temporal compression.

The main contribution of this framework is to connect domains that are usually studied separately: cellular energetics and vascular biology, cognitive event segmentation, episodic memory structure, and subjective long-term time. Rather than suggesting that aging accelerates time itself, the model proposes that aging may alter how densely experience is segmented, encoded, and later reconstructed in memory. In this sense, experienced longevity complements lifespan and healthspan by adding a third aging-relevant question: not only how long life continues and how well biological systems function, but how much experiential structure is retained within that life.

A further strength of the model is its falsifiability. Its central claims can be evaluated by examining whether indirect markers of neuroenergetic reserve are associated with event segmentation, experiential density, and retrospective duration judgments, particularly under conditions of high environmental demand. If such relationships are not observed, the framework should be revised or rejected. If they are, aging science may gain a new line of inquiry into how biological constraints shape the remembered architecture of lived time. Recognizing experienced longevity as a candidate aging-related outcome does not challenge the foundations of geroscience; it extends them.

## Data Availability

The original contributions presented in this study are included in this article/supplementary materials, further inquiries can be directed to the corresponding author.
